# Polyphosphate Attenuates Oxidative Stress to Support Temperature Adaptability in Hot Spring Cyanobacteria

**DOI:** 10.3390/plants15132011

**Published:** 2026-06-29

**Authors:** Xiaohua Song, Yong’an Wei, Minxiang Xu, Di He, Liyu Pan, Chenyu Wang, Jingyun Yin, Chenyuan Kong, Xiaotong Ge, Shunqing Yang, Liuyan Yang, Mengmeng Wang

**Affiliations:** State Key Laboratory of Water Pollution Control and Green Resource Recycling, State Environmental Protection Key Laboratory of Aquatic Ecosystem Health in the Middle and Lower Reaches of the Yangtze River, School of Environment, Nanjing University, Nanjing 210023, China; songxiaohua@smail.nju.edu.cn (X.S.); 522024250099@smail.nju.edu.cn (Y.W.); 522024250113@smail.nju.edu.cn (M.X.); dg21250011@smail.nju.edu.cn (D.H.); 602023250035@smail.nju.edu.cn (L.P.); 18851732535@163.com (C.W.); yinjingyun@smail.nju.edu.cn (J.Y.); 13656655050@163.com (C.K.); gxtocean@163.com (X.G.); dg1925043@smail.nju.edu.cn (S.Y.); yangly@nju.edu.cn (L.Y.)

**Keywords:** thermophilic cyanobacteria, polyphosphate, temperature fluctuations, antioxidant mechanisms, photosynthetic activity

## Abstract

Thermophilic cyanobacteria successfully colonize thermal gradients within hot springs, implying evolved mechanisms to cope with temperature-induced oxidative stress. Although polyphosphate (polyP) is known to contribute to oxidative stress resistance, its specific role in thermophilic cyanobacteria remains poorly understood. To address this, this study established a temperature gradient (30–70 °C) and used phloretin (polyP synthesis inhibitor) plus exogenous polyP to investigate polyP metabolism, redox homeostasis, photosynthetic function, and growth of *Thermosynechococcus* sp. FJSJ-1 from hot spring. The results show that temperature fluctuations specifically induce polyP accumulation, whereas inhibiting polyP synthesis sharply elevates reactive oxygen species (ROS) and overloads intrinsic defenses including superoxide dismutase, catalase, glutathione, and heat shock proteins. Crucially, exogenous polyP rescued phloretin-induced oxidative damage and growth inhibition. PolyP mitigates oxidative damage not by direct ROS scavenging but by integrating and reinforcing endogenous antioxidant network. This protective effect in turn safeguards photosystem II from oxidative attack, thereby preserving photosynthetic pigment stability, phycobiliprotein content, and electron transport efficiency. Taken together, polyP contributes to temperature adaptability in *Thermosynechococcus* sp. FJSJ-1 by coordinating antioxidant defense. This study elucidates a key molecular strategy for thriving across temperature ranges in geothermal ecosystems, advancing microbial adaptation knowledge and providing a theoretical basis for engineering thermotolerant strains for bioremediation and biofuel production.

## 1. Introduction

Microalgae hold substantial biotechnological and environmental potential, with applications spanning industrial wastewater treatment [1], industrial flue gas bioremediation [2], and biofuel production [3,4]. Mesophilic microalgae have been extensively developed for such uses, yet these strains exhibit narrow temperature adaptability [5]. When faced with frequent temperature fluctuations in practical applications—particularly the extreme thermal stress associated with high-temperature industrial wastewater and flue gases—they are susceptible to stress-induced damage, leading to reduced cellular activity, impaired metabolic function, compromised application efficacy, and ultimately limited suitability for complex industrial settings [6,7].

In contrast, thermophilic cyanobacteria are defined as taxa whose optimal growth temperatures exceeds 45 °C [8]. While cyanobacterial oxygenic photosynthesis is widely accepted to function stably up to ~73 °C [9,10], these organisms can withstand extensive temperature variations in geothermal environments. Unlike conventional mesophilic cyanobacteria, thermophilic strains such as *Thermosynechococcus* strains not only tolerate temperature fluctuations but also thrive at elevated temperatures, exhibiting robust CO_2_ fixation and efficient removal of nitrogen, phosphorus, and heavy metals (e.g., Zn, Cd, Pb) from wastewater while producing high-value bioproducts such as thermostable C-phycocyanin [11,12]. This suite of traits endows them with superior stability and biotechnological potential relative to mesophilic microalgae for high-temperature bioprocess applications [13]. The broad temperature adaptability of thermophilic cyanobacteria is underpinned by specialized defensive mechanisms against temperature-induced oxidative stress, and elucidating these mechanisms is pivotal for unlocking the industrial development and utilization of thermophilic cyanobacterial resources.

This exceptional broad temperature adaptability of thermophilic cyanobacteria is rooted in their ancient evolutionary origins. As among the earliest oxygenic photosynthetic organisms on Earth, cyanobacteria emerged in the hyperthermal environments of the Archean Eon and subsequently evolved robust environmental resilience [14,15]. *Thermosynechococcus*—a wide distributed photosynthetic cyanobacterium in hot spring ecosystems—can survive across remarkably broad thermal gradients and has been isolated from diverse geothermal habitats with distinct thermal conditions (Table S1) [16,17,18,19]; temperature heterogeneity even within individual hot spring outlets has driven the evolution of specialized mechanisms to mitigate oxidative stress of varying severity in this genus [20].

Temperature is a key environmental factor governing the survival, metabolism, and distribution of microorganisms, with particularly strong effects on photosynthetic taxa [21]. Core physiological processes such as the synthesis of photosynthetic pigments, carbon fixation and photosynthetic electron transport are highly sensitive to temperature [22]. A central challenge under temperature stress is therefore to maintain the stability of the photosynthetic electron transport chain (PETC) while preserving cellular redox balance [23]. In photosynthetic microbes, elevated temperatures directly impair photosystem II (PSII) and induce reactive oxygen species (ROS) bursts [24], whereas low temperatures suppress metabolic activity and promote oxidative damage [25]. Accordingly, the capacity of thermophilic cyanobacteria to sustain growth across a broad temperature range is likely contingent on a robust, dynamically regulated antioxidant defense system that preserves photosynthetic functionality and cellular redox stability.

Polyphosphate (polyP), a linear or cyclic inorganic polymer composed of tens to hundreds of phosphate units (PO_4_^3−^) linked by high-energy phosphoanhydride bonds, is synthesized by polyphosphate kinase (PPK) and degraded by exopolyphosphatase (PPX) [26,27]. PolyP is present in all three domains of life [28], despite its universal nature, the roles of polyP in cellular metabolism are only beginning to be understood [29]. Beyond its modern cellular functions, polyP is also regarded as a prebiotically plausible, high-energy phosphate reservoir that may have arisen under early-Earth, geothermal conditions [30]. In this view, polyP could have served as a phosphorylating agent and stress-buffering primitive scaffold, potentially conferring an advantage to early (proto)cells facing extreme thermal and nutrient-limiting regimes [30]. In contemporary organisms, polyP contributes to stress adaptation through various mechanisms, such as buffering cellular energy charge, scavenging ROS, chelating metal ions, and regulating protein homeostasis, thereby helping cells cope with environmental challenges [31,32].

PolyP has long been known to affect the ability of a variety of prokaryotic and eukaryotic cells to resist oxidative stress [29]. Early work in *Escherichia coli* showed that deletion of the *ppk* gene increases sensitivity to heat, starvation, and H_2_O_2_, impairing persistence under adverse conditions [33,34]. Subsequent studies of *ppk* mutants in other bacteria, including *Vibrio cholerae* [35], *Lactobacillus* [36], and *Pseudomonas* [37], have consistently demonstrated heightened sensitivity to ROS treatment, further supporting the pivotal role of polyP in bacterial defense against oxidative stress. Besides genetic manipulation, pharmacological inhibition has become an effective strategy for elucidating polyP function. Phloretin, a naturally occurring flavonoid, has been identified as an inhibitor of polyP synthesis. In *Acinetobacter baumannii*, phloretin binds to key residues within the catalytic pocket of PPK1, inducing conformational changes in the enzyme and thereby reducing intracellular polyP accumulation [38]. As a result, bacterial tolerance and persistence under heat stress, oxidative stress, and antibiotic exposure are markedly impaired. A similar inhibitory effect of phloretin on PPK activity has also been reported in *Salmonella* [39]. Furthermore, other small molecules, including gallein [40], etoposide [41], and genistein [41], have been shown to target PPK and attenuate bacterial virulence and stress adaptation through suppression of polyP synthesis. These chemical intervention approaches provide a practical means to study polyP function in dynamic stress responses and in genetically intractable strains.

More importantly, comparative analyses reveal that thermophilic cyanobacteria consistently maintain higher intracellular polyP levels than mesophilic strains [42], strongly implicating polyP metabolism as a central component of their temperature adaptation strategy. Nevertheless, the specific function of polyP in mitigating temperature-induced oxidative stress in thermophilic cyanobacteria remains poorly defined, limiting a comprehensive understanding of their adaptation physiology. To address this gap, we investigated the role of polyP in the oxidative stress response of a thermophilic cyanobacterium across a temperature gradient. We hypothesized that these microorganisms employ a polyP-centered regulatory network that coordinates energy balance, antioxidant defense, and photosynthetic protection to sustain growth under varying geothermal temperatures. Elucidating this mechanism will not only advance our understanding of how thermophilic cyanobacteria colonize high-temperature springs but may also inform strategies for engineering microbial systems for high-temperature applications, such as bioremediation or photosynthetic bioproduction.

## 2. Materials and Methods

### 2.1. Thermophilic Cyanobacterial Cultivation

The thermophilic cyanobacterium strain FJSJ-1, used in this study, was isolated from a hot spring in Shajian Town, Zhangzhou City, Fujian Province, China (24.72° N, 117.56° E). The sampling site had an in situ temperature of 52 °C and a pH range of 6.5–7. Taxonomic identification via 16S rRNA gene sequencing confirmed the isolate belongs to the genus *Thermosynechococcus* (Figure S1), and it was therefore designated *Thermosynechococcus* sp. FJSJ-1.

After isolation, the strain was purified through repeated streaking, followed by microscopic selection of single colonies and serial dilution cultures to eliminate contaminants until an axenic culture was obtained. The purified strain was subsequently inoculated into BG-11 (pH 7) liquid medium and cultured in a light incubator at 52 °C under a photoperiod of 12 h light to12 h dark, with an illumination intensity of 30 μmol photons·m^−2^·s^−1^.

### 2.2. Mechanistic Exploration of PolyP Function in Broad Temperature Adaptation

#### 2.2.1. PolyP Synthesis Inhibition

Phloretin was applied to suppress intracellular polyP synthesis [38]. Thermophilic cyanobacterial cultures in the logarithmic phase were briefly sonicated to disperse cell aggregates into single cells, and the resulting culture was then equally aliquoted into 500 mL Erlenmeyer flasks at an initial density of 0.18 g·L^−1^ biomass. Three treatments were established: blank control (no addition), solvent control (0.5% DMSO, added to facilitate phloretin dissolution and to match solvent conditions across groups), and phloretin treatment (0.5% DMSO + 350 mg·L^−1^ phloretin). Each group was incubated at different temperatures (30 °C, 40 °C, 50 °C, 60 °C, 70 °C) with five biological replicates. Following a 5-day culture period, biomass, photosynthetic parameters, different forms of phosphorus contents, and the activities of antioxidant enzymes—PPK and PPX—were quantified.

#### 2.2.2. Exogenous PolyP Addition

To verify the role of polyP in alleviating temperature stress, logarithmic-phase thermophilic cyanobacterial cultures were equally aliquoted into flasks containing either control (no addition) or polyP treatment (2 mg·L^−1^ sodium hexametaphosphate). The same temperature regimes and biological replicates as described previously were applied. After cultivation, biomass, photosynthetic parameters, and ROS levels were quantified.

#### 2.2.3. Combined Phloretin, PolyP, and Inorganic Phosphate Treatments

To evaluate the contribution of polyP to the physiological responses of *Thermosynechococcus* under phloretin treatment, and to distinguish its effects from those of phosphate nutrition alone, a combined treatment experiment was conducted at 50 °C. Thermophilic cyanobacterial cultures in the logarithmic growth phase were distributed into 500 mL Erlenmeyer flasks at an initial biomass density of 0.10 g·L^−1^. Seven experimental groups were established: blank control (no addition), solvent control (0.5% DMSO), phloretin (0.5% DMSO + 350 mg·L^−1^ phloretin), phloretin + polyP (0.5% DMSO + 350 mg·L^−1^ phloretin + 2 mg P·L^−1^ sodium hexametaphosphate), phloretin + PO_4_^3−^ (0.5% DMSO + 350 mg·L^−1^ phloretin + 2 mg P·L^−1^ NaH_2_PO_4_), polyP (2 mg·L^−1^ sodium hexametaphosphate); PO_4_^3−^ (2 mg P·L^−1^ NaH_2_PO_4_). All cultures were incubated at 50 °C under identical light and shaking conditions, with five biological replicates per treatment. After a 5-day cultivation period, intracellular ROS, antioxidant enzyme activities, heat shock protein (HSP) levels, phycobiliprotein content, and biomass were quantified.

#### 2.2.4. In Vitro Added PolyP Removal of ROS

To examine whether polyP directly contributes to oxidative stress mitigation, we assessed its ability to scavenge ROS in vitro O_2_^−^ and H_2_O_2_. Superoxide scavenging was determined using the pyrogallol autoxidation assay. A mixture of pyrogallol (0.2 mL, 10 mM) and Tris-HCl buffer (9.8 mL, pH 7.5) served as the control. Absorbance at 320 nm was recorded every 30 s for 10 min. For the polyP treatment, pyrogallol (0.2 mL, 10 mM) was combined with Tris-HCl buffer (9.7 mL, pH 7.5) and sodium polyP solution (0.1 mL, 1 g·L^−1^). Reaction rates were calculated from the slope of absorbance versus time.

To evaluate the direct scavenging of H_2_O_2_, sodium polyP solution (0.1 mL, 1 g·L^−1^) was added to H_2_O_2_ (10 mL, 1 mM) and incubated for 2 h at 20 °C. A control was prepared by replacing polyP with ultrapure water. Residual H_2_O_2_ concentrations were measured after incubation in both groups.

O_2_^−^ and H_2_O_2_ were measured using kits from Solarbio (Beijing, China). Detailed experimental procedures, calculation formulas, and quality controls are provided in Supplementary Materials.

### 2.3. Physiological and Biochemical Parameters Determination

#### 2.3.1. Biomass Quantification

The thermophilic cyanobacterial culture was thoroughly mixed, and a 50 mL aliquot was filtered onto pre-weighed 0.45 μm glass fiber filters (Delvstlab, Jiaxing, China) that had been dried at 105 °C to constant weight [43]. After filtration, the filters were dried at 60 °C to constant weight. Biomass concentration (g·L^−1^) was calculated from dry weight relative to the sample volume.

#### 2.3.2. Photosynthetic Parameters Measurement

A 3 mL aliquot of cyanobacterial culture was dark-adapted for 2 min before measurements. The maximum photochemical efficiency of PSII (F_v_/F_m_), the actual photochemical quantum yield (Yield), and maximum electron transport rate (ETR_m_) were determined using a Water-PAM II chlorophyll fluorometer (WALZ, Effeltrich, Germany) [44]. ETR_m_ was derived by fitting electron transport rates (calculated across a gradient of actinic light intensities) to a non-rectangular hyperbola model. Calculations followed standard definitions:

F_v_/F_m_ = (F_m_ − F_o_)/F_m_ (1)

Yield = (F_m_′ − F_s_)/F_m_′ (2)

where F_o_ and F_m_ are the minimum and maximum fluorescence after dark adaptation, F_s_ and F_m_′ are the steady-state and maximum fluorescence under actinic light.

For Chlorophyll *a* (Chl *a*) and carotenoid determination, 10 mL well-mixed culture was filtered and homogenized on ice in 90% acetone. Extracts were centrifuged at 6000× *g* for 10 min at 4 °C, and supernatant absorbance was measured at 450, 630, 645, 663, and 750 nm. Pigment concentrations were calculated using Equations (3) and (4) [45,46]:

Chl *a* (mg·L^−1^) = 11.64 × A_663_ − 2.16 × A_645_ + 0.10 × A_630_ − 9.58 × A_750_ (3)

Carotenoids (mg·L^−1^) = 4.1 × A_450_ − 0.553 × A_663_ + 0.118 × A_645_ (4)

For phycobiliprotein quantification, cells were disrupted by ultrasonication on ice at 60% amplitude (3 s pulses with 7 s intervals for 5 min), followed by centrifugation at 8000× *g* for 10 min at 4 °C. As *Thermosynechococcus* sp. FJSJ-1 does not contain phycoerythrin (PE) [47], a two-component spectrophotometric method was employed for the quantification of phycocyanin (PC) and allophycocyanin (APC), using only absorbance readings at 620 nm and 650 nm [48]:

PC (mg·mL^−1^) = (A_620_ − 0.7 × A_650_)/7.38 (5)

APC (mg·mL^−1^) = (A_650_ − 0.19 × A_620_)/5.65 (6)

#### 2.3.3. Quantification of PolyP Content

PolyP was extracted from 50 mL cultures following a modified phenol/chloroform protocol [42]. Briefly, cells were lysed with 2% trichloroacetic acid (TCA), and pellets were resuspended in EDTA solution and extracted with phenol/chloroform (1:1, *v*/*v*). Supernatants were treated with RNase A and DNase I at 37 °C for 1.5 h, followed by a second phenol/chloroform extraction. PolyP was precipitated by transferring the supernatant into pre-chilled tubes containing ethanol and sodium acetate (pH 5.3), incubating at −20 °C for 1.5 h, washing with 70% ethanol, and air-drying to obtain polyP granules, which were further processed according to Zhu et al. [49]. Intracellular polyP granules were visualized using metachromatic granule staining, and changes in their size distribution were examined microscopically.

#### 2.3.4. PPK and PPX Activities Analysis

The thermophilic cyanobacterial culture of 10 mL was centrifuged at 6000× *g* for 10 min at 4 °C, and then the pellet was resuspended in pre-chilled phosphate-buffered saline (PBS, 50 mM, pH 6.8). The thermophilic cyanobacterial cells were disrupted on ice using a probe sonicator (Nanjing Xianou Instruments Manufacture Co., Ltd., Nanjing, China; 20 kHz, 60% amplitude) with 3 s pulses alternating with 7 s intervals for a total of 5 min (30% duty cycle). The probe tip was immersed 1.5 cm below the liquid surface. Lysates were centrifuged again at 10,000× *g* for 10 min at 4 °C, and the supernatant was collected and kept on ice for further assays. Enzyme activities of PPK (JZT-926217O1) and PPX (JZT-925717O1) were determined using commercial ELISA kits (Jiaozi Teng, Nanjing, China) according to the manufacturer’s instructions. Briefly, standards and samples were loaded into pre-coated wells, followed by sequential incubation with enzyme conjugates and substrates. After termination, absorbance was measured at 450 nm, and enzyme activities of PPK and PPX were calculated from standard curves and expressed as U·mg^−1^ protein.

#### 2.3.5. Antioxidant System and Biochemical Parameters Analysis

The preparation method of intracellular extracts was performed with reference to the procedures for PPK and PPX assays. The contents of total protein (A045-3-2), ATP (A095-1-1), reduced glutathione (GSH) (A006-1-1) and the activities of superoxide dismutase (SOD) (A001-3-2) and catalase (CAT) (A007-1-1) were quantified using commercial kits from Nanjing Jiancheng Bioengineering Institute (Nanjing, China). HSP activity (YJ034759) was determined with ELISA kits from mlbio (Shanghai, China). All analyses were performed strictly according to the manufacturers’ protocols with five biological replicates. Detailed experimental procedures, calculation formulas, and quality controls are provided in Supplementary Materials.

### 2.4. Data Processing and Statistical Analysis

All data were expressed as mean ± standard deviation (SD, n = 5). Normality of distribution was assessed using the Shapiro–Wilk test, and homogeneity of variances was verified by Levene’s test prior to analysis. Statistical analyses were performed using Origin 2026 (OriginLab, Northampton, MA, USA). One-way ANOVA followed by Tukey’s multiple comparisons test was used to evaluate significant differences among the five treatment groups. Differences were considered significant at *p* < 0.05.

## 3. Results and Discussion

### 3.1. PolyP-Mediated Thermophilic Cyanobacterial Growth Across Temperature Fluctuations

Clarifying the temperature adaptation characteristics of the thermophilic cyanobacterium *Thermosynechococcus* sp. FJSJ-1, this study measured its growth rate across five temperature gradients (30–70 °C) and fitted the data using the Cardinal model (Table 1). The fitted model indicated a theoretical growth temperature range of 22.71–69.19 °C and an optimal growth temperature of 51.87 °C. This optimal temperature closely matches the environmental temperature of the strain’s isolation in hot spring (52 °C), refining that *Thermosynechococcus* sp. FJSJ-1 has evolved a temperature-adapted physiology precisely aligned with its native hot-spring niche, which likely underpins its ecological competitiveness.

**Figure 1:**
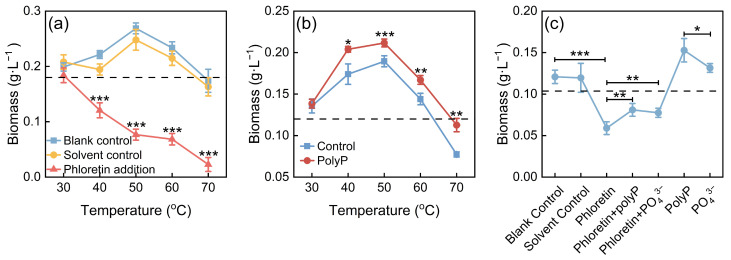
Effects of temperature and phloretin supplementation on the growth of *Thermosynechococcus* sp. FJSJ-1 after 5 days of culture. (**a**,**b**) Growth performance under temperature gradients in phloretin-treated and exogenous polyP-supplemented groups; (**c**) Biomass changes under phloretin treatment with exogenous polyP or orthophosphate supplementation at 50 °C. The dashed line represents the initial biomass level for each group. Panels (**a**–**c**) were derived from independent experiments using separate pre-cultured cell stocks; while absolute biomass values under the 50 °C control differed modestly between batches, the relative trends regarding phloretin suppression and polyP rescue remained fully consistent. Data represent mean ± SD of biological replicates. Asterisks indicate statistical significance between the phloretin-treated group and blank control or between the polyP-supplemented group and control (* *p* < 0.05, ** *p* < 0.01, *** *p* < 0.001).

To directly probe the specific role of polyP in this broad-temperature adaptation, we employed a pharmacological intervention strategy using phloretin. A concentration of 350 mg·L^−1^ was determined to suppress intracellular polyP synthesis by over 80% (Figure S2). The solvent control (DMSO) showed no significant effect on growth, confirming that the observed phenotypes were specifically attributable to polyP depletion rather than DMSO. To further exclude non-specific toxic effects of phloretin, we performed a rescue experiment by co-administering phloretin with exogenous polyP. The results showed that polyP significantly alleviated the biomass decreases that probably result from cell rupture, whereas supplementation with an equimolar amount of inorganic phosphate was less effective (Figure 1c). These findings indicate that polyP depletion, rather than broad-spectrum toxicity, largely accounts for the biomass loss induced by phloretin. Upon polyP inhibition, *Thermosynechococcus* sp. FJSJ-1 exhibited clear growth defects across 30–70 °C, with markedly large decreases in biomass compared to the blank control group (Figure 1a). These results demonstrate that polyP availability is critical for maintaining robust growth and biomass accumulation under temperature fluctuation, consistent with the pleiotropic, stress-related deficits reported for *ppk* disruption in thermophilic cyanobacteria [42]. In addition, recent studies indicate that polyP influences the spatial organization of carboxysomes and the carbon-concentrating mechanism in cyanobacteria [50]. Therefore, its loss would be expected to reduce carbon-fixation efficiency under energy-constrained conditions that accompany high-temperature growth, potentially exacerbating the growth defects observed here.

Conversely, supplementation with exogenous polyP significantly enhanced biomass accumulation across all temperatures tested (Figure 1b). It is worth noting that the biomass accumulation promoted by exogenous polyP was significantly higher than that induced by supplementation with an equimolar amount of inorganic phosphate (Figure 1c), indicating that polyP reserves not only suffice for basal tolerance but can actively bolster cellular buffering capacity against temperature fluctuations. This effect suggests that the role of polyP is not limited to providing a phosphate source, it also enhances the cell’s temperature adaptability through its unique biochemical functions. The growth-promoting role of polyP appears evolutionarily conserved, as similar phenomena have been reported in higher plants where polyP application promotes root growth in wheat under phosphorus deficiency or salt stress [51,52]. Even in non-photosynthetic systems, polyP directly promotes fibroblast mitotic activity [53]. Therefore, these results demonstrate that polyP is a key factor in maintaining the normal growth of thermophilic cyanobacteria across a broad temperature range.

### 3.2. PolyP Metabolism-Mediated Homeostasis

Given the critical role of polyP in supporting the broad-temperature growth of *Thermosynechococcus* sp. FJSJ-1, we further investigated its underlying metabolic regulatory mechanisms. Our results indicate that the dynamic equilibrium of intracellular polyP is fundamental to its temperature-buffering function.

**Figure 2:**
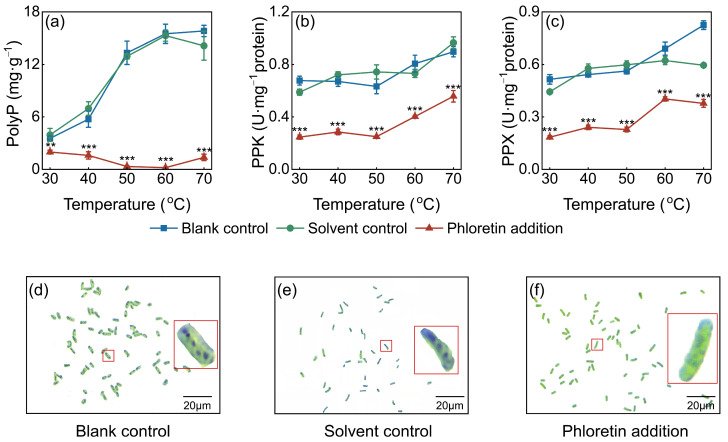
Effects of temperature on (**a**) polyphosphate (polyP)-phosphorus content, (**b**) polyphosphate kinase (PPK) activity, (**c**) exopolyphosphatase (PPX) activity, and (**d**–**f**) microscopic observation of intracellular polyP granules (50 °C) in *Thermosynechococcus* sp. FJSJ-1 with phloretin added or not. The red square in (**d**–**f**) indicates the region magnified inset, which shows a magnified view of polyP granules. Data are shown as mean ± SD (n = 5). Asterisks indicate statistical significance between the phloretin-treated group and the blank control (** *p* < 0.01, *** *p* < 0.001).

Temperature gradients substantially reshaped intracellular polyP metabolism in *Thermosynechococcus* sp. FJSJ-1. Under normal growth conditions, polyP content exhibited a continuous gradient across 30–70 °C (Figure 2a), reflecting a metabolically flexible system that modulates polyP flux in response to ambient temperature and thereby serves as an intrinsic buffer against temperature fluctuations. This temperature-dependent accumulation was regulated by the activities of key synthesis and degradation enzymes. Both PPK and PPX activities in *Thermosynechococcus* sp. FJSJ-1 increased markedly with rising temperature (Figure 2b,c), consistent with the general acceleration of enzymatic reaction rates under increased thermal kinetic energy [54]. This trend paralleled the observed polyP accumulation, suggesting that *Thermosynechococcus* sp. FJSJ-1 preserves polyP reserves through basal activity at 30–40 °C while enhancing synthesis at 50–70 °C, thereby maintaining metabolic equilibrium across temperature gradients.

This adaptive strategy appears evolutionarily conserved. For instance, polyP accumulation upon warming has been observed in mesophilic microalgae [55], Arctic *Cylindrocystis* [56], and cryophilic ice worm *Mesenchytraeus solifugus* [57], whereas low temperatures stimulate polyP accumulation in *Eiseniella andrei* [57]. Moreover, polyP levels exhibit diverse trends in response to temperature shifts among different *Acinetobacter* strains [58]. Taken together, these observations suggest that the reprogramming of polyP metabolism is a universal biological mechanism for adaptation to varying temperatures, underscoring its pivotal role in growth across broad temperature ranges.

When polyP synthesis was inhibited, its levels remained significantly lower than that of the control group at all temperatures (43.9–98.7% reduction; Figure 2a), consistent with the reduction in polyP granules observed by staining (Figure 2d–f), confirming that pharmacological inhibition effectively depletes the overall polyP reserve. Corresponding enzyme assays showed that phloretin treatment significantly suppressed PPK activity at all temperature conditions (Figure 2b), indicating that phloretin acts as an effective inhibitor, directly or indirectly impairing the catalytic function of PPK and thereby blocking polyP biosynthesis—a finding consistent with earlier reports [38]. Concurrently, PPX activity also exhibited a declining trend with phloretin treatment (Figure 2c). This decrease is likely attributable to substrate-limitation (reduced polyP availability) rather than direct enzyme inhibition [59,60], which aligns with the phosphorous metabolism disorder phenotype observed in *Escherichia coli ppk* mutants [61]. Together, these results reinforce that maintaining the synthesis and turnover equilibrium of polyP constitutes a core regulatory process enabling thermophilic cyanobacteria to acclimate to wide temperature variations.

### 3.3. PolyP Attenuates Temperature-Induced Oxidative Stress

**Figure 3:**
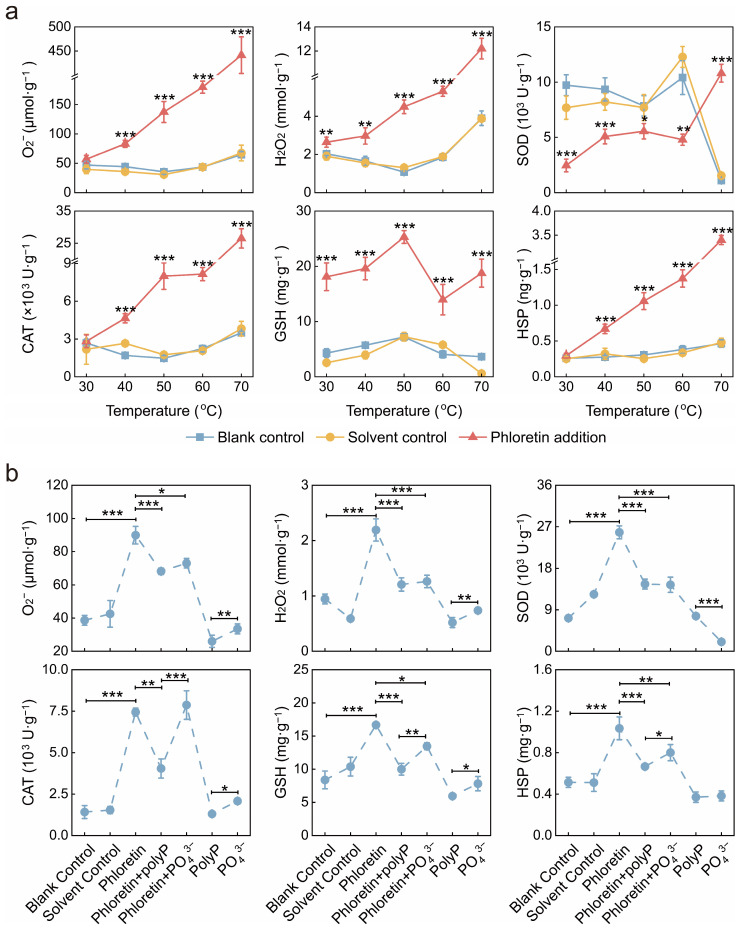
Oxidative stress parameters and defense responses of *Thermosynechococcus* sp. FJSJ-1 under phloretin treatment with varying temperatures (**a**) or with exogenous polyP/orthophosphate (**b**) supplementation at 50 °C. Data represent mean ± SD of biological replicates. Asterisks indicate statistical significance between the phloretin-treated group and the blank control (* *p* < 0.05, ** *p* < 0.01, *** *p* < 0.001).

Oxidative stress is a major physiological challenge for cyanobacteria exposed to temperature-heterogeneous environments. PolyP plays a pivotal role in overcoming this challenge by serving as a core regulator of antioxidant defenses, enabling the maintenance of oxidative homeostasis across a broad temperature spectrum. Inhibition of polyP synthesis significantly elevated ROS generation (O_2_^−^ and H_2_O_2_) of *Thermosynechococcus* sp. FJSJ-1 across broad temperature ranges (Figure 3a), indicating that polyP deficiency broadly impairs the ability of cells to maintain redox homeostasis at different temperature, thereby exacerbating oxidative stress. In response to the elevated ROS, cells upregulated the activity of antioxidant enzymes (SOD, CAT), synthesized increased amounts of reduced GSH, and markedly induced the expression of HSPs (Figure 3a). HSP expression is closely linked to cellular ROS levels [62], as ROS can directly modify HSPs structure and activity, and also regulate HSP expression through redox-signaling pathways [63]. Thus, polyP inhibition appears to amplify the ROS levels, which in turn stimulates HSP expression as part of a compensatory stress-mitigation response.

To exclude the nutritional effect of exogenous phosphorus supplementation, we co-administered phloretin with equimolar polyP or inorganic phosphate. The results showed that polyP supplementation significantly reduced ROS levels and alleviated the overactivation of antioxidant enzymes and HSPs, whereas inorganic phosphate only partially mitigated these effects and was markedly less effective than polyP (Figure 3b). Under normal culture conditions, the same comparative result further supported this conclusion (Figure 3b). Therefore, the regulation of oxidative homeostasis by polyP is not simply attributable to phosphorus supplementation but rather reflects a specific antioxidant function inherent to polyP itself. This is consistent with a previous report that in bloom-forming cyanobacteria, polyP not only alleviates oxidative damage but also maintains intracellular phosphate homeostasis under heatwaves and alkaline stress [64].

**Figure 4:**
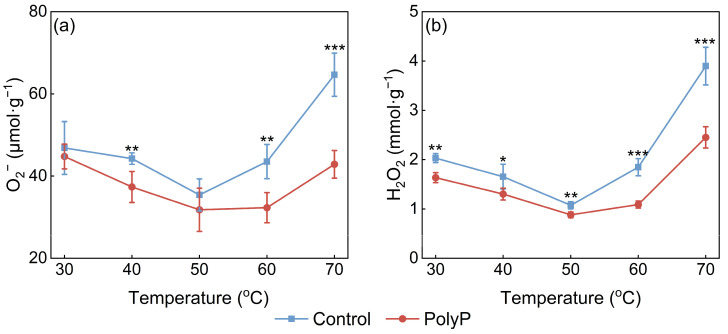
Effects of temperature on oxidative markers contents of (**a**) O_2_^–^ and (**b**) H_2_O_2_ in *Thermosynechococcus* sp. FJSJ-1 in exogenous polyP-supplemented group. Data represent mean ± SD of biological replicates. Asterisks indicate statistical significance between polyP-supplemented group and the control (* *p* < 0.05, ** *p* < 0.01, *** *p* < 0.001).

Further studies show that exogenous application of polyP effectively reduced ROS levels across the entire temperature range of 30–70 °C (Figure 4), confirming its role as a universal redox regulator supporting the thermophilic cyanobacterial survival across temperature zones. PolyP achieved O_2_^–^ scavenging rates of 2.3–39.07% and H_2_O_2_ reduction rates of 4.60–66.37%, indicating that its ability to dynamically modulate effects according to oxidative stress intensity at different temperatures, thereby adapting to physiological demands across the temperature fluctuations. The antioxidant function of polyP appears to be evolutionarily conserved across diverse autotrophic lineages, as demonstrated in transgenic rice overexpressing *ppk* [49] and in wheat treated with polyphosphate fertilizer [65]. This functional conservation extends even to animal cells, as probiotic-derived polyP has been shown to reduce lipopolysaccharide-induced ROS accumulation in macrophages [66]. Importantly, in vitro assays demonstrated that polyP itself does not directly scavenge ROS (Figure S3), ruling out chemical quenching as the mechanism. Instead, its protective effects appear to depend on the coordinated regulation of various physiological processes within the cell. Mechanistically, polyP enhances antioxidant defense through four complementary pathways: (i) Stabilizing antioxidant enzymes as a molecular chaperone, preventing irreversible denaturation [67,68]; (ii) Chelating transition metals (Fe^2+^, Cu^+^) to suppress Fenton chemistry and hydroxyl radical formation [27,69,70,71]; (iii) Maintaining Mn^2+^ homeostasis to support Mn-based ROS scavenging [72,73]; and (iv) Serving as a high-energy phosphate reservoir that sustains ATP regeneration via PPK, fueling repair processes such as protein refolding and glutathione recycling [74,75]. Collectively, these findings suggest that polyP contributes to the attenuation of temperature-induced oxidative damage.

### 3.4. PolyP Protects the Photosynthesis System

**Figure 5:**
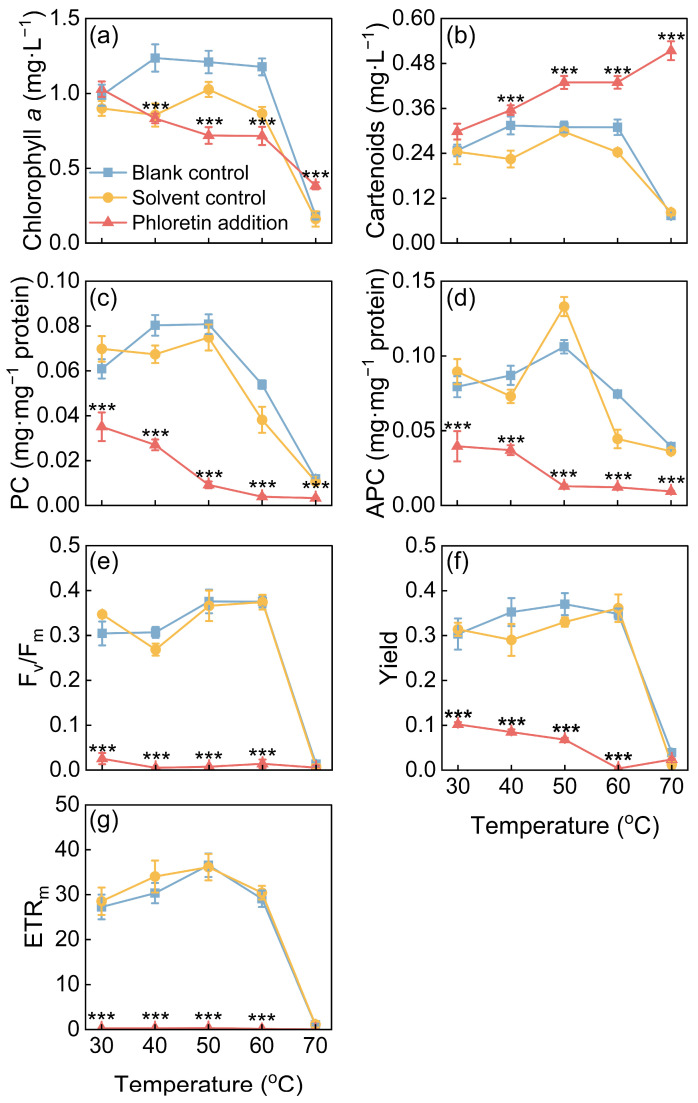
Effects of temperature on photosynthetic performances of (**a**) Chlorophyll *a* (Chl *a*), (**b**) carotenoids, (**c**) phycocyanin (PC), (**d**) allophycocyanin (APC), (**e**) F_v_/F_m_, (**f**) Yield, and (**g**) ETR_m_ in *Thermosynechococcus* sp. FJSJ-1 in phloretin-treated groups, respectively. Data represent mean ± SD of biological replicates. Asterisks indicate statistical significance between the phloretin-treated group and the blank control (*** *p* < 0.001).

Having established that polyP alleviates cellular oxidative stress, we next examined whether this protective effect extends to the photosynthetic apparatus, a primary target of temperature-induced damage. The photosynthetic machinery—pigments, lipids, structural proteins, and enzymes—is highly sensitive to temperature fluctuations [76]. While low temperatures inhibit its activity, high temperatures may lead to component degradation. In cyanobacteria, temperature-induced ROS can further damage these components, thereby impairing photosynthetic efficiency [77]. Consistent with this, exposure to sub- and supra-optimal temperatures, significantly reduced Chl *a*, and phycobiliprotein levels, as well as key photosynthetic efficiency parameters (Figure 5). These findings are consistent with previous research indicating that temperature deviations from optimum impede electron transport in the PETC, resulting in photosystem damage [78]. At 30–40 °C, suppressed electron transport results in sustained oxidative pressure [79]. In contrast, temperatures above the optimum inflict direct damage on photosynthetic components, provoking an acute ROS burst [80]. Both pathways converge to disrupt the integrity of the photosynthetic apparatus, collectively representing the principal challenges to maintaining photosynthetic function across a wide temperature range.

When polyP synthesis was inhibited, Chl *a* and phycobiliprotein contents declined at all temperatures (Figure 5a,c,d), accompanied by suppressed ATP production (Figure S4). To verify the relationship between polyP depletion and photosynthetic damage, we supplemented exogenous polyP on the basis of phloretin-inhibited endogenous polyP synthesis. The results showed a significant recovery in phycobiliprotein content and photosynthetic parameters (Figure S5), confirming that the inhibition of endogenous polyP is indeed one of the key factors contributing to the instability of the photosynthetic apparatus. The damage likely arises from combined effects of ROS accumulation and disrupted metal-ion homeostasis. PolyP is known to chelate Mg^2+^ (the central ion of chlorophyll) and Ca^2+^ (a structural cofactor of PSII), thereby its absence may therefore destabilize chlorophyll–protein complexes and aggravate disassembly the oxygen-evolving complex [81,82,83]. Concurrently, the decrease in the F_v_/F_m_, along with declines in the Yield and ETR_m_ (Figure 5e–g), points to impaired electron transport and structural damage to PSII caused by accumulated ROS [76,84].

In contrast, carotenoids levels increased across all temperatures in polyP-deficient cells, with the extent of the increase correlating with the corresponding rise in ROS load (Figure 5b). As light-harvesting pigments and antioxidants, carotenoids are critical for photoprotection. This pattern indicates an emergency activation of compensatory antioxidant defense, in which excessive ROS production triggered upregulation of carotenoids biosynthesis to enhance ROS scavenging and temporarily protect photosystem [85]. This shift in pigment composition represents a metabolic trade-off, diverting resources from light-harvesting to photoprotection in response to the elevated ROS load implied by the compromised photosynthetic parameters. Similar compensatory upregulation has been reported in thermophilic *Synechococcus* sp. OS-B’, where ATP shortage and electron transport chain uncoupling caused ROS bursts and carotenoids accumulation [86].

**Figure 6:**
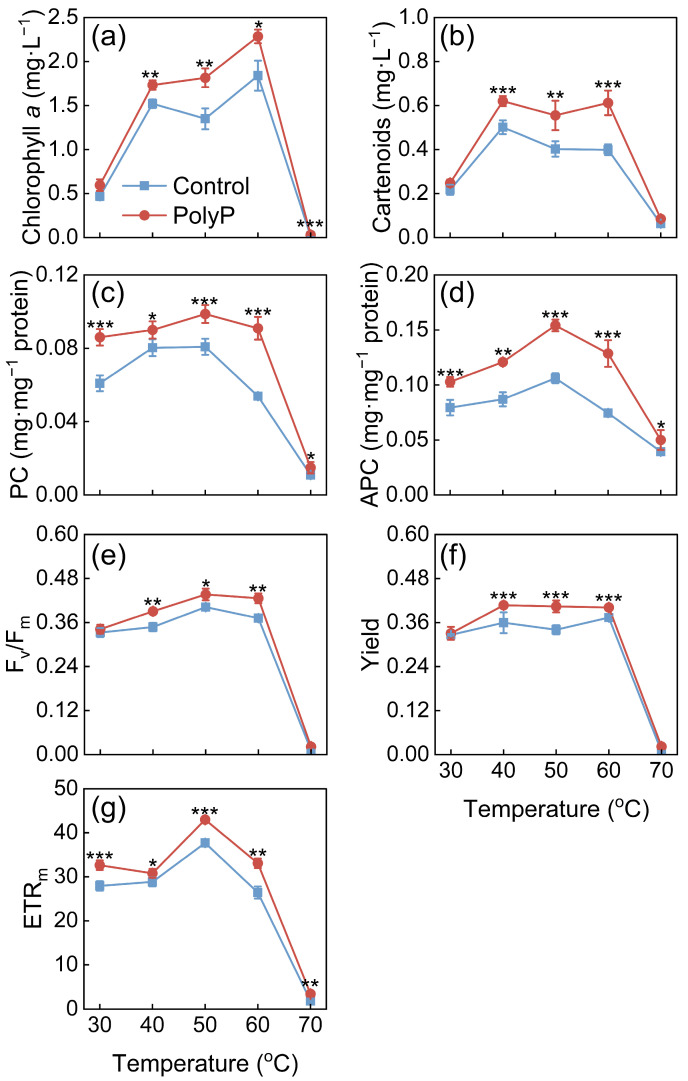
Effects of temperature on photosynthetic performances of (**a**) Chlorophyll *a* (Chl *a*), (**b**) carotenoids, (**c**) phycocyanin (PC), (**d**) allophycocyanin (APC), (**e**) F_v_/F_m_, (**f**) Yield, and (**g**) ETR_m_ in *Thermosynechococcus* sp. FJSJ-1 in exogenous polyP-supplemented group. Data represent mean ± SD of biological replicates. Asterisks indicate statistical significance between polyP-supplemented group and the control (* *p* < 0.05, ** *p* < 0.01, *** *p* < 0.001).

Conversely, supplementation with exogenous polyP enhances Chl *a*, phycobiliprotein, and photosynthetic efficiency parameters at all temperatures (Figure 6a,c–g). Notably, under normal culture conditions, the promoting effect of polyP was markedly stronger than inorganic phosphate (Figure S5), indicating that polyP possesses a specific regulatory function beyond merely providing phosphate nutrition. This protective effect likely results from two complementary mechanisms: (i) Improved nutrient uptake and pigment biosynthesis, consistent with reports that polyP application in durum wheat enhanced N, K, and P acquisition and promoted chlorophyll synthesis [87]; And (ii) polyP-mediated redox regulation that shields photosynthetic components from oxidative degradation [88,89]. PolyP also acts as a molecular chaperone to stabilize photosynthetic protein folding and maintain PSII reaction center integrity [68]. These findings align with those showing that polyP protects photosynthetic proteins from photo-oxidative damage in *Microcystis* [90].

The ability of polyP to preserve photosynthetic function appears to be an evolutionarily conserved strategy. The application of polyP elevated PSII efficiency (F_v_/F_m_) in foliar-sprayed wheat [87], and enhanced PSII donor-side electron transport in chickpea [91], further indicating that polyP-mediated preservation of photosystem functionality is an evolutionarily conserved strategy for maintaining energy metabolism under temperature stress. Similar to the phenomenon observed in *Tetraselmis marina* where carotenoids accumulation occurs under sufficient phosphorus conditions [92], the application of polyphosphate in this study also promoted carotenoids synthesis (Figure 6b). This effect may be attributed to the enhancement of energy metabolism and photosynthetic system activity by polyphosphate, as well as its synergistic regulatory role within the overall antioxidant network.

Thus, polyP stabilizes photosynthetic pigments and phycobiliproteins, restores photosynthetic electron transport efficiency, and ensures efficient light energy capture and conversion, thereby supporting the photosynthetic autotrophic growth of thermophilic cyanobacteria across a wide temperature range.

### 3.5. PolyP-Associated Physiological Responses Underlying Temperature-Range Adaptability

**Figure 7:**
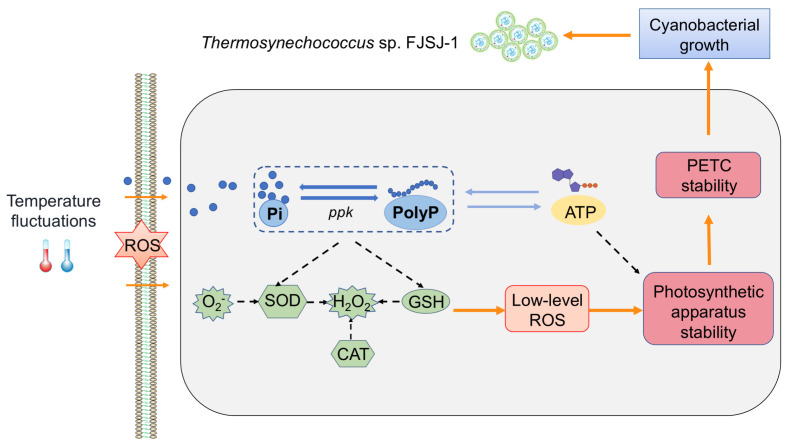
Conceptual framework illustrates how temperature-regulated polyphosphate (polyP) metabolism supports temperature adaptation in thermophilic cyanobacteria. Deviations from the optimal temperature elevate reactive oxygen species (ROS) levels. PolyP accumulation, regulated by polyphosphate kinase (PPK) dynamics, enhances endogenous antioxidant networks and stabilizes photosynthetic electron transport chain, thereby maintaining energy balance and enabling sustained growth across the 30–70 °C gradient. Solid lines indicate material metabolic flows, dashed lines represent regulatory interactions, and orange arrows denote the ultimate biological effects.

Based on these findings, we propose an integrated model highlighting the potential regulatory role of polyP and its association with the ability of a thermophilic cyanobacterium to maintain growth across a broad temperature range (Figure 7). Temperature fluctuations represent a key growth-limiting factor, as they induce oxidative stress and impair the photosynthetic apparatus. In response, cells dynamically modulate their polyP metabolic pool. Temperature signals directly enhance the activity of the PPK enzyme, driving the synthesis and accumulation of polyP. The accumulated polyP then mitigates oxidative stress by reinforcing the antioxidant network and stabilizes the photosynthetic apparatus, thereby maintaining electron transport and ATP synthesis. Ultimately, through the synergistic effect of oxidative stress alleviation and energy supply assurance, polyP maintains cell growth under various temperature conditions.

We conducted exogenous polyP rescue and orthophosphate control experiments to verify the functional significance of polyP in the proposed model and to exclude potential pleiotropic effects of phloretin. The results showed that phloretin-induced oxidative damage and growth inhibition could be significantly rescued by exogenous polyP, whereas orthophosphate at an equivalent phosphorus concentration only partially alleviated growth and pigment losses associated with phosphorus starvation. These findings indicate that the function of polyP cannot be replaced by phosphate alone, and its role in wide-temperature adaptation extends beyond merely serving as a phosphorus source. Specifically, polyP acts as a phosphorus reservoir under stress and, through its molecular chaperone function and metal chelation activity, serves as a macromolecular antioxidant that provides unique protection not mimicked by orthophosphate ions. This antioxidant function renders polyP irreplaceable in protecting against oxidative stress induced by temperature fluctuations, particularly under high temperatures. Given the natural competence of this strain, exogenously supplied polyP may be partially internalized and contribute to intracellular homeostasis [93]. Therefore, the protective effects observed upon polyP supplementation may arise from combined extracellular and intracellular actions. Nevertheless, we cannot entirely exclude the possibility that phloretin may have minor polyP-independent effects under prolonged treatment. Therefore, we plan to use a ppk mutant for genetic validation in future studies to further clarify this effect.

In addition to oxidative stress regulation, polyP has also been reported to participate in modulating carboxysome spatial organization and the CO_2_-concentrating mechanism in cyanobacteria. Previous studies have shown that polyP depletion disrupts nucleoid architecture and carboxysome positioning, thereby reducing carbon fixation efficiency and imposing carbon limitation [50]. This mechanism likely operates in parallel with the oxidative stress pathway, jointly restricting biomass accumulation under polyP-deficient conditions. Future studies could further distinguish the relative contributions of oxidative stress and carboxysome positioning/carbon fixation limitation to the polyP-depletion phenotype.

The elucidation of this regulatory mechanism not only deepens the understanding of environmental adaptation in thermophilic life but also lays a critical theoretical foundation for the engineered application of thermophilic cyanobacteria. The capacity for growth across a wide temperature range accommodates fluctuating temperature conditions in industrial settings, enabling direct utilization of waste heat, flue gas CO_2_, and nutrient-rich wastewater for biomass production. These organisms combine carbon fixation with tolerance to heavy metals and metabolism of organic pollutants, allowing concurrent treatment of complex industrial effluents during cultivation. This integration of carbon sequestration and bioremediation offers a practical route for converting waste streams into feedstocks for biomaterials and value-added natural products.

In turn, the enhancement of broad temperature tolerance and biotechnological efficacy in thermophilic cyanobacteria via polyP-targeted strain engineering offers a straightforward and practical optimization strategy for their large-scale industrial implementation. Collectively, the broad-temperature-range growth characteristics of thermophilic cyanobacteria establish them as an exemplary microbial resource for environmental bioremediation and the industrialization of green biotechnologies. Our elucidation of the regulatory mechanisms governing broad-temperature-range growth in these cyanobacteria further consolidates the theoretical framework underpinning their industrial deployment, accelerating the translation of native thermophilic cyanobacterial strains into engineered, application-ready microbial chassis.

## 4. Conclusions

This study demonstrates that polyP acts as a central hub enabling thermophilic cyanobacteria to maintain growth across a broad temperature gradient typical of hot spring habitats. Across 30–70 °C, temperature fluctuations consistently increased oxidative pressure, whereas polyP accumulation mitigated this stress by reinforcing the endogenous antioxidant network rather than directly scavenging ROS. Inhibiting polyP synthesis amplified ROS accumulation and disrupted enzymatic (SOD, CAT) and non-enzymatic (e.g., GSH and photoprotective pigments) defenses, leading to impaired photosynthetic integrity and reduced biomass accumulation, while exogenous polyP supplementation restored redox balance, stabilized PSII performance, and promoted sustained growth across a broad temperature range. Collectively, polyP integrates multiple protective roles: It buffers phosphorus homeostasis, stabilizes photosystems, preserves light-harvesting components, and reinforces the antioxidant network. Thus, by orchestrating these functions, polyP establishes an adaptive “metabolism–structure–redox” framework that underlies the capacity of thermophilic cyanobacteria to thrive across temperature-variable heterogeneous environments. These findings not only elucidate a key molecular strategy for ecological success in extreme habitats but also provide a theoretical foundation for engineering thermally tolerant strains for biotechnology applications.

## Figures and Tables

**Table 1:** Growth rates of *Thermosynechococcus* sp. FJSJ-1 across a temperature gradient and the corresponding Cardinal model fitting parameters.

Temperature (°C)	Growth Rate (g·L^−1^·d^−1^)	Parameter	Fitted Value
30	0.0038	T_min_ ^1^	22.71
40	0.0083	T_opt_ ^2^	51.87
50	0.0178	T_max_ ^3^	69.19
60	0.0107	R^2^	0.93
70	−0.0012	/ ^4^	/

^1^ T_min_: The minimum growth temperature; ^2^ T_opt_: The optimum growth temperature; ^3^ T_max_: The maximum growth temperature; ^4^ /: No value.

## Data Availability

The original contributions presented in this study are included in the article/Supplementary Materials. Further inquiries can be directed to the corresponding authors.

## Supplementary Materials

The following supporting information can be downloaded at: https://www.mdpi.com/article/10.3390/plants15132011/s1, Text S1: Determination of ROS; Text S2: Determination of antioxidant system and biochemical parameters; Figure S1: The phylogenetic tree constructed based on the 16S rRNA sequence; Figure S2: The effect of phloretin concentration on the polyP content of *Thermosynechococcus* sp. FJSJ-1; Figure S3: In vitro effects of polyP on ROS scavenging O_2_^−^ (a) and H_2_O_2_ (b) measured in cell-free assays with polyP addition; Figure S4: Effects of temperature on contents of ATP in *Thermosynechococcus* sp. FJSJ-1 with phloretin added or not; Figure S5: Phycobiliprotein content of *Thermosynechococcus* sp. FJSJ-1 under phloretin treatment with exogenous polyP or orthophosphate (PO_4_^3−^) supplementation at 50 °C; Table S1: Distribution of *Thermosynechococcus* in hot springs of different temperatures worldwide [11,18,94,95,96,97,98,99,100,101,102,103,104,105].

## Abbreviations

The following abbreviations are used in this manuscript:

PETC	Photosynthetic electron transport chain
ROS	Reactive oxygen species
PolyP	Polyphosphate
PPK	Polyphosphate kinase
PPX	Exopolyphosphatase
H_2_O_2_	Hydrogen peroxide
O_2_^–^	Superoxide anions
PSII	Photosystem II
Chl *a*	Chlorophyll *a*
PC	Phycocyanin
APC	Allophycocyanin
PE	Phycoerythrin
TP	Total phosphorus
Pi	Inorganic phosphate-phosphorus
TCA	Trichloroacetic acid
GSH	Glutathione
SDO	Superoxide dismutase
CAT	Catalase
HSP	Heat shock proteins
