# Supplementary material for: Polyphosphate Attenuates Oxidative Stress to Support Temperature Adaptability in Hot Spring Cyanobacteria
Source: Plants (Basel). 2026 Jun 29;15(13):2011. doi: 10.3390/plants15132011 (PMC13363987; doi:10.3390/plants15132011)
Supplement: Supplementary file 1 [file plants-15-02011-s001.zip › plants-4337128-supplementary.pdf]

Polyphosphate Attenuates Oxidative Stress to Support Temperature Adaptability in Hot Spring Cyanobacteria

Xiaohua Song, Yong'an Wei, Minxiang Xu, Di He, Liyu Pan, Chenyu Wang, Jingyun Yin, Chenyuan Kong, Xiaotong Ge, Shunqing Yang, Liuyan Yang, Mengmeng Wang*

State Key Laboratory of Water Pollution Control and Green Resource Recycling, State Environmental Protection Key Laboratory of Aquatic Ecosystem Health in the Middle and Lower Reaches of the Yangtze River, School of Environment, Nanjing University, Nanjing 210023, Jiangsu, China

*Corresponding author:

Email: wangmm@nju.edu.cn (Mengmeng Wang)

Supplementary Text S1 Determination of ROS

Superoxide anion (O_2^-)

The content of O_2^- was determined using the commercial assay kit (Cat. No.: BC1295, Solarbio, Beijing, China) strictly according to the manufacturer's instructions. Briefly, 10 mL of thermophilic cyanobacterial culture was centrifuged at 6,000 × g for 10 min at 4°C, and the pellet was resuspended in 1 mL of extraction buffer and thoroughly homogenized. The homogenate was then centrifuged at 13,000 × g for 20 min at 4°C, and the resulting supernatant was collected for subsequent analysis.

For the assay, blank, determination, and standard tubes were prepared with five biological replicates per group. The reaction mixtures were assembled as follows: 100 μL extraction buffer was added to the blank tube; 40 μL cyanobacterial supernatant plus 60 μL extraction buffer was added to the determination tube; and 40 μL standard

solution plus 60 μL extraction buffer was added to the standard tube. Subsequently, 80 μL of Reagent I was added to each tube, mixed thoroughly, and incubated in a water bath at 37°C for 20 min. Following this, 60 μL of Reagent II and 60 μL of Reagent III were sequentially added, mixed well, and incubated at 37°C for an additional 20 min. Finally, 100 μL of chloroform (Reagent IV) was added to each tube, vortexed, and centrifuged at 6,000 × g for 5 min at 25°C to separate the phases. Then, 200 μL of the upper aqueous phase was carefully transferred to a 96-well plate, and the absorbance was measured at 530 nm using a microplate reader. The O_2^- concentration was calculated based on the standard curve.

Hydrogen peroxide (H_2O_2)

The content of H_2O_2 was determined using the commercial assay kit (Cat. No.: H930426, Solarbio, Beijing, China) according to the manufacturer's protocol. Briefly, 10 mL of thermophilic cyanobacterial culture was centrifuged at 6,000 × g for 10 min at 4°C. The resulting pellet was resuspended in 1 mL of pre-chilled acetone and disrupted by ultrasonication at 20% amplitude (3 s pulses alternating with 10 s intervals, 30 cycles). The lysate was centrifuged at 8,000 × g for 10 min at 4°C, and the supernatant was maintained on ice for subsequent assays.

Reagent I was dissolved completely in 3 mL of concentrated hydrochloric acid immediately before use. Reagents I, II, and III were pre-incubated in a water bath at 25°C for 10 min prior to the assay. For the colorimetric reaction, control and determination tubes were prepared with five biological replicates per group. Specifically, 250 μL of acetone was mixed with 25 μL of Reagent I and 50 μL of

Reagent II in the control tube, whereas 250 μL of cyanobacterial supernatant was mixed with 25 μL of Reagent I and 50 μL of Reagent II in the determination tube. The mixtures were vortexed thoroughly and centrifuged at 4,000 × g for 10 min at room temperature. The supernatant was carefully discarded, and the precipitate was retained. Subsequently, 250 μL of Reagent III was added to dissolve the precipitate. After mixing and standing at room temperature for 5 min, 200 μL of the resulting solution was transferred to a 96-well microplate, and the absorbance was measured at 415 nm using a microplate reader.

The H_2O_2 content in the cyanobacterial sample was calculated using the formula for cell samples provided in the kit:

$$H_2O_2 \text{ content } (\mu mol \cdot g^{-1}) = 2.67 \times (A_{determination} - A_{control}) / W$$

Where W is the weight of the cyanobacterial sample (g).

Supplementary Text S2 Determination of antioxidant system and biochemical parameters

Total protein

Total protein content was determined using a bicinchoninic acid (BCA) protein assay kit (Catalog No. A045-3, Nanjing Jiancheng Bioengineering Institute, Nanjing, China). Briefly, cell pellets were washed with ice-cold phosphate-buffered saline (PBS, 50 mM, pH 6.8) and lysed on ice using a probe sonicator (20 kHz, 60% amplitude) with 3 s pulses alternating with 7 s intervals for a total of 5 min (30% duty cycle). The lysed was centrifuged at 10,000 × g for 10 min at 4°C, and the supernatant was collected for analysis.

For the assay, 20 μL of sample supernatant or bovine serum albumin (BSA)

standard was mixed with 250 μL of BCA working reagent in a 1.5 mL centrifuge tube. After vortexing, the mixture was incubated at 37ºC for 30 min, followed by the addition of 750 μL of 5-fold diluted Reagent III (provided in the kit). The tube was vortexed again and allowed to stand at room temperature for 5 min. Subsequently, 200 μL of the reaction mixture was transferred to a 96-well microplate, and the absorbance was measured at 562 nm using a microplate reader.

Protein concentration was calculated according to the following formula:

$$\text{Protein concentration } (\mu g \cdot mL^{-1}) = (A_{sample} - A_{blank}) / (A_{standard} - A_{blank}) \times C_{standard} \times N$$

Where $C_{standard}$ is the concentration of the BSA standard, and N is the dilution factor of the sample.

ATP content

ATP content was determined using a colorimetric assay kit (Catalog No. A095-1-1, Nanjing Jiancheng Bioengineering Institute, Nanjing, China). Cell pellets were washed twice with ice-cold double-distilled water and resuspended in 500 μL of the same. Cells were disrupted on ice using a probe sonicator (20 kHz, 60% amplitude) with 3 s pulses alternating with 7 s intervals for a total of 5 min (30% duty cycle). The lysate was boiled for 10 min, followed by vortexing for 1 min and centrifugation at 3,500 × g for 10 min at 4ºC to obtain the supernatant for analysis.

To avoid phosphorus contamination, disposable plastic tubes were used throughout. Four types of tubes—blank, standard, determination, and control—were prepared with five biological replicates per group. For the determination tube, 30 μL of sample supernatant was mixed with 100 μL of Reagent I, 200 μL of Reagent II, and 30

μL of Reagent III. For the control tube, Reagent III was replaced with 30 μL of double-distilled water. For the standard tube, 30 μL of ATP standard (1 mmol·L^{-1}) was substituted for the sample supernatant. For the blank tube, 30 μL of double-distilled water was substituted for the sample supernatant, and Reagent III was omitted. All tubes were vortexed thoroughly and incubated in a water bath at 37°C for 30 min.

Subsequently, 500 μL of Reagent IV was added to each tube. After mixing, the tubes were centrifuged at 4,000 × g for 5 min, and 300 μL of the supernatant from each was transferred to a clean tube. Then, 500 μL of chromogenic application solution (Reagent V) was added, mixed well, and allowed to stand at room temperature for 2 min. This was followed by the addition of 500 μL of Reagent VI (terminator), mixing, and incubation at 37°C for 5–10 min. Finally, 200 μL of the reaction mixture was transferred to a 96-well microplate, and the absorbance was measured at 636 nm using a microplate reader.

ATP concentration was calculated according to the following formula:

$$\text{ATP content } (\mu\text{mol}\cdot g^{-1}\text{prot}) = (A_{determination} - A_{control}) / (A_{standard} - A_{blank}) \times C_{standard} \times N / Cpr$$

Where $C_{standard}$ is the concentration of the ATP standard (1000 μmol·L^{-1}); N is the sample dilution factor, and Cpr is the protein concentration of the cyanobacterial supernatant (gprot·L^{-1}).

Reduced glutathione (GSH)

The content of GSH was determined using a commercial kit (Cat. No.: A006-1-1, Nanjing Jiancheng Bioengineering Institute, Nanjing, China) according to the

manufacturer's instructions. Briefly, 10 mL of thermophilic cyanobacterial culture was centrifuged at 6,000 × g for 10 min at 4ºC. The resulting pellet was resuspended in ice-cold PBS (50 mM, pH 6.8) and disrupted on ice using a probe sonicator (20 kHz, 60% amplitude) with 3 s pulses alternating with 7 s intervals for a total of 5 min (30% duty cycle). The lysate was centrifuged at 10,000 × g for 10 min at 4°C. The supernatant (0.4 mL) was mixed with an equal volume of Reagent I working solution, vortexed thoroughly, and centrifuged at 4,000 × g for 10 min. The resulting supernatant was collected for the chromogenic reaction.

Blank, standard, and determination tubes were prepared with five biological replicates per group. For the blank tube, 0.8 mL of Reagent I was used; for the standard tube, 0.8 mL of GSH standard (20 $\mu mol \cdot L^{-1}$); and for the determination tube, 0.8 mL of the pretreated supernatant. Subsequently, 1.0 mL of Reagent II, 200 μL of Reagent III, and 40 μL of Reagent IV were sequentially added to each tube. After vortexing, the mixture was allowed to stand at room temperature for 5 min. Then, 200 μL of the reaction mixture was transferred to a 96-well microplate, and the absorbance was measured at 420 nm using a microplate reader.

The GSH content was calculated using the following formula:

$$GSH\ (mg \cdot g^{-1}) = (A_{determination} - A_{blank}) / (A_{standard} - A_{blank}) \times C_{standard} \times 307 / W \times V$$

Where $C_{standard}$ is the concentration of the GSH standard (20 $\mu mol \cdot L^{-1}$); N is the sample dilution factor; W is the weight of the cyanobacterial sample (g), and V is the total volume of the sample during homogenization or disruption with Reagent I working solution.

Superoxide dismutase (SOD)

The activity of SOD was determined using a WST-1 assay kit (Cat. No.: A001-3, Nanjing Jiancheng Bioengineering Institute, Nanjing, China), according to the manufacturer's instructions. Briefly, 10 mL of thermophilic cyanobacterial culture was centrifuged at 6,000 × g for 10 min at 4ºC, and the pellet was resuspended in PBS (50 mM, pH 6.8). Cyanobacterial cells were disrupted on ice using a probe sonicator (20 kHz, 60% amplitude) with 3 s pulses alternating with 7 s intervals for a total of 5 min (30% duty cycle). The lysate was centrifuged at 10,000 × g for 10 min at 4°C, and the collected supernatant was retained for the assay.

Working solutions were prepared immediately before use: substrate working solution was prepared by mixing Reagent II (substrate stock) and Reagent I (buffer) at a ratio of 1:200 (v/v); enzyme working solution was prepared by mixing Reagent III (enzyme stock) and Reagent IV (dilution buffer) at a ratio of 1:10 (v/v).

For the assay, four types of wells were prepared in a 96-well microplate with five biological replicates per group: (i) control well: 20 μL distilled water + 20 μL enzyme working solution; (ii) control blank well: 20 μL distilled water + 20 μL enzyme dilution buffer; (iii) determination well: 20 μL appropriately diluted supernatant + 20 μL enzyme working solution; and (iv) determination blank well: 20 μL appropriately diluted supernatant + 20 μL enzyme dilution buffer. Subsequently, 200 μL of substrate working solution was added to each well using a multi-channel pipette. The plate was gently shaken to ensure thorough mixing and incubated at 37ºC for 20 min. The absorbance was then measured at 450 nm using a microplate reader.

The SOD activity was calculated using the following formula:

$$\text{SOD inhibition rate (\%)} = [1 - (A_{\text{control}} - A_{\text{control blank}}) / (A_{\text{determination}} - A_{\text{determination blank}})] \times 100\%$$

$$\text{SOD } (U \cdot g^{-1}) = \text{SOD inhibition rate} / 50\% \times N \times 12 / W \times V$$

Where N is the sample dilution factor; W is the weight of the cyanobacterial sample (g), and V is the volume of the homogenization buffer (mL).

Catalase (CAT)

The activity of CAT was determined using the ammonium molybdate method (Cat. No.: A007-1-1, Nanjing Jiancheng Bioengineering Institute, Nanjing, China) according to the manufacturer’s instructions. Briefly, 10 mL of thermophilic cyanobacterial culture was centrifuged at 6,000 × g for 10 min at 4ºC, and the pellet was resuspended in ice-cold PBS (50 mM, pH 6.8). Cyanobacterial cells were disrupted on ice using a probe sonicator (20 kHz, 60% amplitude) with 3 s pulses alternating with 7 s intervals for a total of 5 min (30% duty cycle). The lysate was centrifuged at 10,000 × g for 10 min at 4ºC, and the collected supernatant was retained for the assay.

Reagents I and II were pre-warmed at 37ºC for 5 min prior to the assay. For each sample, determination and control tubes were prepared with five biological replicates. For the determination tube, 100 μL of supernatant was mixed with 1.0 mL of Reagent I (substrate buffer) and pre-incubated at 37ºC for 3–5 min. Subsequently, 100 μL of Reagent II (H_2O_2 substrate) was added to initiate the reaction, mixed immediately, and incubated at 37ºC for exactly 60 s. The reaction was terminated immediately by the addition of 1.0 mL of Reagent III (acidic ammonium molybdate solution), followed by

thorough mixing. Then, 100 μL of Reagent IV was added and mixed well. For the control tube, 1.0 mL of Reagent I was incubated with 100 μL of Reagent II for exactly 60 s at 37°C, followed by sequential addition of 1.0 mL of Reagent III and 100 μL of Reagent IV; finally, 100 μL of supernatant was added to account for background absorbance. Subsequently, 200 μL of the reaction mixture from each tube was transferred to a 96-well microplate, and the absorbance was measured at 405 nm using a microplate reader.

The calculation was performed using the formula provided in the kit:

$$CAT\ (U \cdot g^{-1}) = (A_{control} - A_{determination}) \times 271 / V_1 / W \times V_2$$

Where V_1 is the sample volume in this reaction system (0.1 mL); W is the weight of the cyanobacterial sample (g), and V_2 is the volume of the homogenization buffer (mL).

Heat shock protein (HSP)

HSP content was determined using a sandwich ELISA kit (mlbio, Shanghai, China) according to the manufacturer's protocol. Briefly, blank, standard, and sample wells were prepared. Standards (50 μL) were added to standard wells, while 10 μL of sample diluted with 40 μL of sample dilution buffer was added to sample wells. Blank wells received 50 μL of dilution buffer only. The plate was incubated at 37°C for 30 min and then washed five times with wash buffer. Subsequently, 50 μL of HRP-conjugated reagent was added to standard and sample wells (blank wells excluded), followed by incubation at 37°C for another 30 min. After five additional washes, 50 μL of chromogen solution A and 50 μL of chromogen solution B were sequentially added to

each well. The plate was gently mixed and incubated at 37ºC in the dark for 15 min. Finally, 50 μL of stop solution was added to terminate the reaction, and the absorbance was measured at 450 nm using a microplate reader.

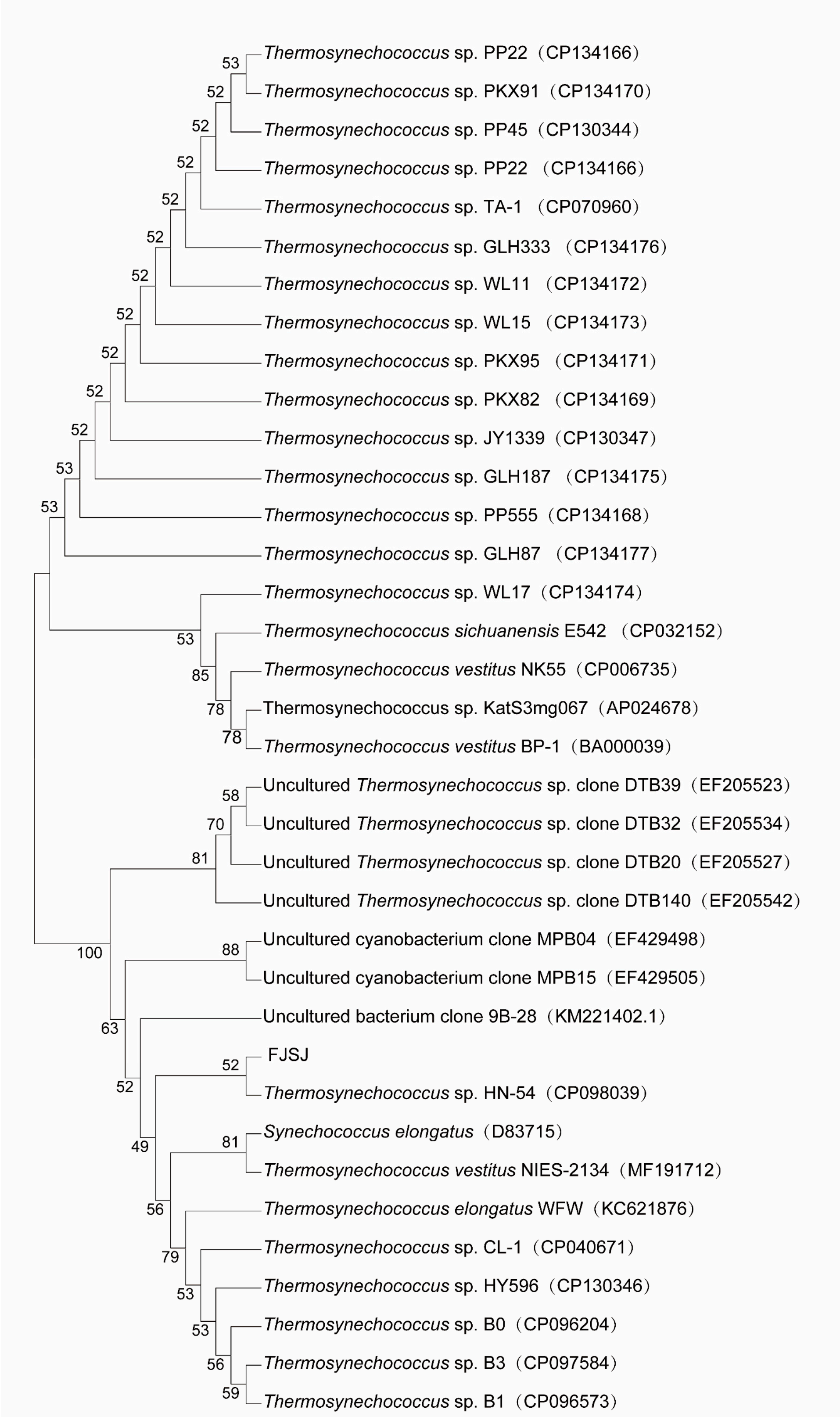

Supplementary Figure S1. The phylogenetic tree constructed based on the 16S rRNA sequence.

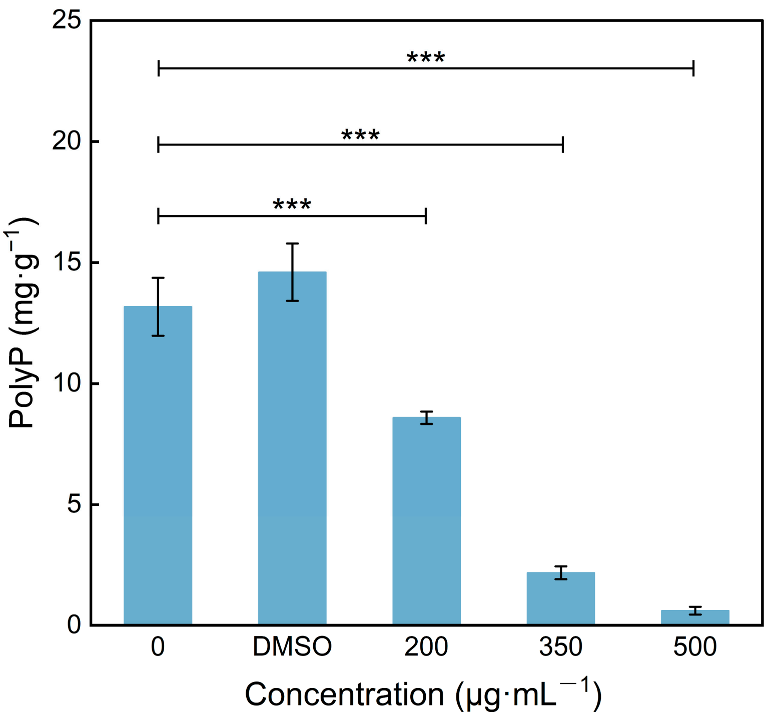

Supplementary Figure S2. The effect of phloretin concentration on the polyP content of *Thermosynechococcus* sp. FJSJ-1. Data represent mean ± SD of biological replicates. Asterisks indicate statistical significance (*$p < 0.05$, **$p < 0.01$, ***$p < 0.001$).

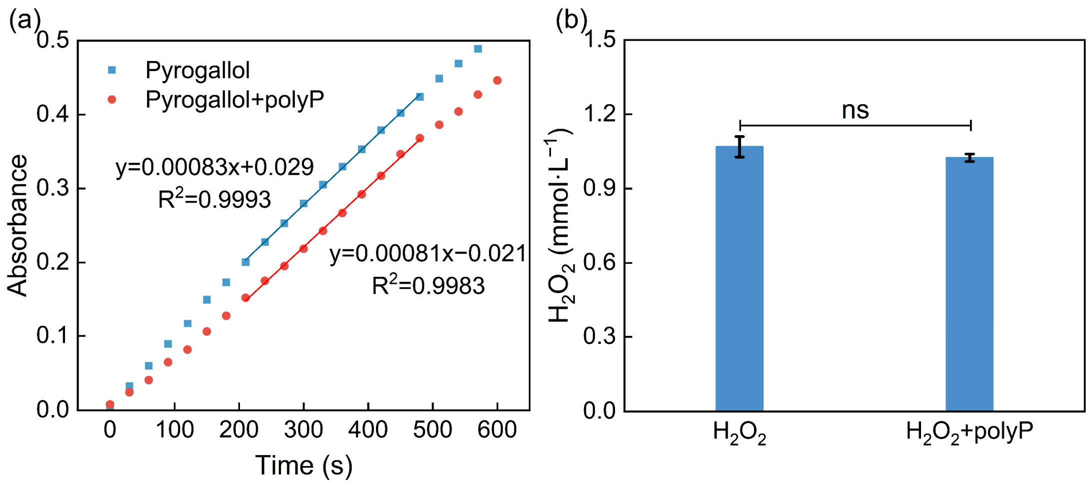

Supplementary Figure S3. In vitro effects of polyP on ROS scavenging O_2^- (a) and H_2O_2 (b) measured in cell-free assays with polyP addition. Data represent mean ± SD of biological replicates.

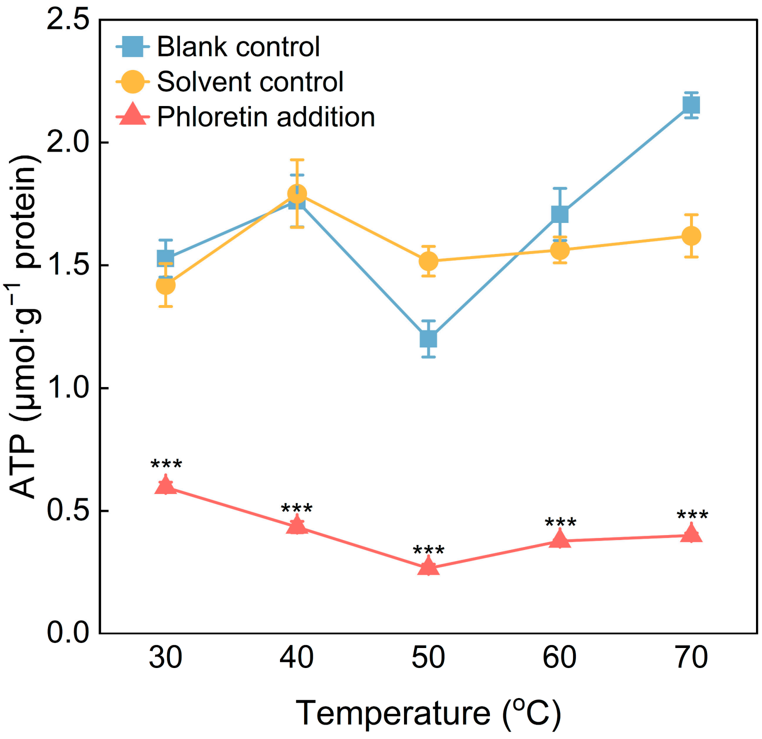

Supplementary Figure S4. Effects of temperature on contents of ATP in *Thermosynechococcus* sp. FJSJ-1 with phloretin added or not. Data are shown as mean ± SD (n = 5). Asterisks indicate statistical significance between the phloretin-treated group and the blank control (*$p < 0.05$, **$p < 0.01$, ***$p < 0.001$).

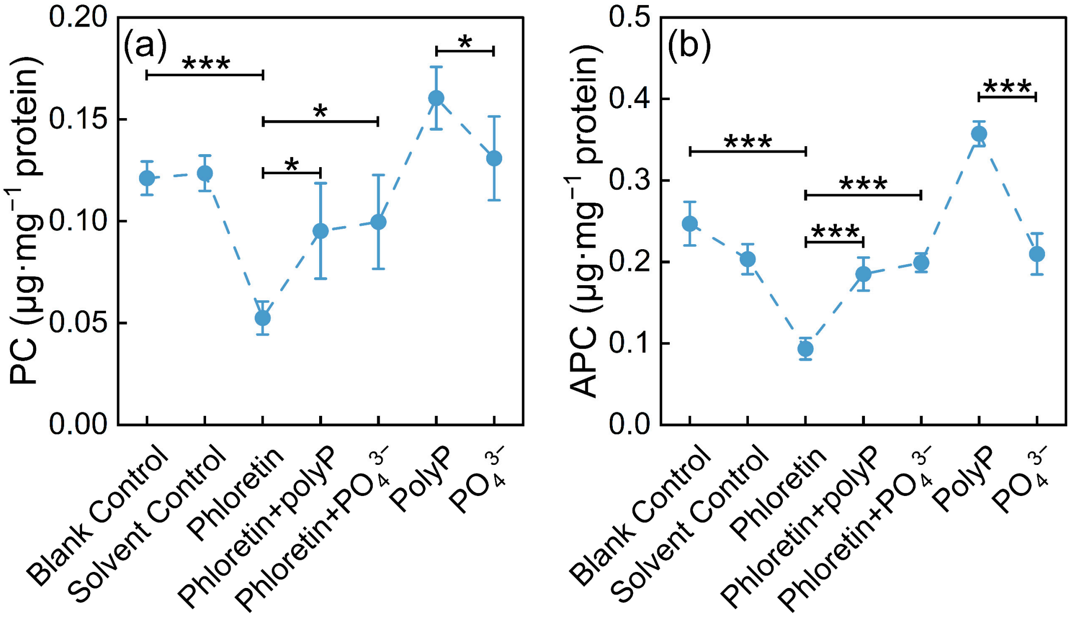

Supplementary Figure S5. Phycobiliprotein content of *Thermosynechococcus* sp. FJSJ-1 under phloretin treatment with exogenous polyP or orthophosphate (PO_4^{3-}) supplementation at 50ºC. (a)phycocyanin, (b) allophycocyanin. Data represent mean ± SD. Asterisks indicate statistical significance (**p* < 0.05, ***p* < 0.01, ****p* < 0.001).

Supplementary Table S1. Distribution of *Thermosynechococcus* in hot springs of different temperatures worldwide.

Hot spring	Location	Temperature	Strains	References
Jinata hot spring	Shikinejima, Tokyo, Japan	37–46ºC	*Thermosynechococcus* sp. M3746_W2019_013<br>*Thermosynechococcus* sp. J003	[94]
Taian hot spring	Taiwan，China	40–63ºC	*Thermosynechococcus elongatus* TA-1	[95]
Okuoku-hachikurou hot spring	Akita, Japan	44ºC	*Thermosynechococcus* sp. OHK43	[96]
Shivlinga hot spring	Ladhak, India	46ºC	*Thermosynechococcus* sp. M46_R2017_013	[97]
Ganzi Prefecture	Sichuan Province, China	41.1–95ºC	*Thermosynechococcus elongatus* PKUAC	[98]
Nakabusa hot spring	Nagano Prefecture，Japan	52–60ºC	*Thermosynechococcus sp.* NK55a	[99]
Yunomine hot spring	Wakayama，Japan	53–55ºC	*Thermosynechococcus vestitus* NIES-2134	[100]
Nakabusa hot spring	Nagano Prefecture，Japan	56-64ºC	Thermosynechococcus sp. NK_OTU_003	[101]
Tattapani hot spring	Tattapani, India	55ºC	*Thermosynechococcus* sp. M55_K2018_012	[102]
Beppu hot spring	Kyushu，Japan	55ºC	*Thermosynechococcus elongatus* BP-1	[103]

Sembawang hot spring	Singapore National Parks，Singapore	61.4℃	*Thermosynechococcus elongatus*	[104]
Chin-Lun hot spring	Taiwan，China	62ºC	*Thermosynechococcus* sp. CL-1	[105]
Lianhua Lake hot spring	Sichuan, China	67.2ºC	*Thermosynechococcus elongatus* PKUAC-SCTE542	[11]
Nakabusa hot spring	Nagano，Japan	76ºC	*Thermosynechococcus* sp. NK55a	[99]
Yunomine hot spring	Wakayama, Japan	91ºC	*Thermosynechococcus vestitus* NIES-2178	[18]
Tattapani hot spring	Tattapani, India	98℃	*Thermosynechococcus* sp. M98_K2018_005	[102]
